# Concurrent brain-responsive and vagus nerve stimulation for treatment of drug-resistant focal epilepsy

**DOI:** 10.1016/j.yebeh.2022.108653

**Published:** 2022-03-16

**Authors:** Mesha-Gay Brown, Stefan Sillau, Danielle McDermott, Lia D. Ernst, David C. Spencer, Dario J. Englot, Hernán F.J. González, Proleta Datta, Ioannis Karakis, Danielle Becker, John D. Rolston, Amir Arain, Vikram R. Rao, Michael Doherty, Alexandra Urban, Cornelia Drees

**Affiliations:** a University of Colorado, Department of Neurology, United States; b Oregon Health & Science University, Department of Neurology, United States; c Vanderbilt University, Department of Biomedical Engineering, United States; d Vanderbilt University Medical Center, Department of Neurological Surgery, United States; e University of Nebraska Medical Center, Department of Neurology, United States; f Emory University School of Medicine, Department of Neurology, United States; g University of Pennsylvania, Department of Neurology, United States; h University of Utah, Departments of Neurosurgery and Biomedical Engineering, United States; i University of Utah, Department of Neurology, United States; j University of California, San Francisco, Department of Neurology and Weill Institute for Neurosciences, United States; k Swedish Neuroscience Institute, Department of Neurology, Seattle, United States; l University of Pittsburgh, Department of Neurology, United States; m Mayo Clinic Arizona, Department of Neurology, United States; n Centura Health Physician Group, Neuroscience and Spine, CO, United States

**Keywords:** Drug-resistant epilepsy, Responsive neurostimulation, Vagus nerve stimulation, Neuromodulation, Seizure outcome, Complications

## Abstract

**Objective:**

Clinical trials of a brain-responsive neurostimulator, RNS^®^ System (RNS), excluded patients with a vagus nerve stimulator, VNS^®^ System (VNS). The goal of this study was to evaluate seizure outcomes and safety of concurrent RNS and VNS stimulation in adults with drug-resistant focal-onset seizures.

**Methods:**

A retrospective multicenter chart review was performed on all patients with an active VNS and RNS who were treated for a minimum of 6 months with both systems concurrently. Frequency of disabling seizures at baseline before RNS, at 1 year after RNS placement, and at last follow-up were used to calculate the change in seizure frequency after treatment. Data on adverse events and complications related to each device were collected.

**Results:**

Sixty-four patients from 10 epilepsy centers met inclusion criteria. All but one patient received RNS after VNS. The median follow-up time after RNS implantation was 28 months. Analysis of the entire population of patients with active VNS and RNS systems revealed a median reduction in seizure frequency at 1 year post-RNS placement of 43% with a responder rate of 49%, and at last follow-up a 64% median reduction with a 67% responder rate. No negative interactions were reported from the concurrent use of VNS and RNS. Stimulation-related side-effects were reported more frequently in association with VNS (30%) than with RNS (2%).

**Significance:**

Our findings suggest that concurrent treatment with VNS and RNS is safe and that the addition of RNS to VNS can further reduce seizure frequency.

## 1. Introduction

The efficacy of brain-responsive neurostimulation with the RNS System [NeuroPace, Inc, Mountain View, CA] (RNS) has been well established through prospective clinical trials, with a 68% median reduction in seizure frequency at 6 years and 75% at 9 years [1]. However, due to a Food and Drug Administration (FDA) requirement, these clinical trials excluded patients that were actively treated with a VNS System [LivaNova, Inc, London, United Kingdom] (VNS). More recently, a study of real-world outcomes with the RNS demonstrated similar effectiveness in a shorter timeframe with a median reduction in seizure frequency of 67% at 1 year and 75% at 2 years, but again this study did not look at use of RNS in patients with active VNS [2]. One study examined the electrographic effects of VNS stimulation on recorded RNS electrocorticography (ECoG) providing support for the theory of desynchronization as VNS mechanism of action, but clinical outcomes in these patients with combined use of the two systems were not reviewed [3].

There are now three FDA-approved neurostimulation devices available for treatment of drug-resistant focal epilepsy: VNS, RNS, and deep-brain stimulation (DBS) [4,5]. Intrinsic characteristics of these devices differ widely, but all are considered palliative. There are no head-to-head trials that establish their relative efficacy, and there are no established methods that predict which device would be most effective for a given patient. Thus, analogous to selection of antiseizure medications (ASMs), the choice of a particular neurostimulation device for a given patient remains largely empiric.

For patients whose seizures do not respond optimally to one device, it has not been investigated – due to FDA restrictions in studies preceding device approval – whether added, concurrent use of other devices is safe and beneficial, or if failure of the initial therapy predicts failure of added subsequent therapies. Indeed, in clinical practice multiple neurostimulation systems have already been implanted into individual patients [3,6], but the paucity of systematically collected outcome data in these patients critically limits our ability to counsel patients with the most treatment-resistant epilepsy regarding outcomes with dual - or triple - stimulation therapy and best possible combinations of devices.

Vagus nerve stimulation was approved in 1997 and, although beneficial for many patients, those whose seizures do not respond to VNS may go on to try other therapies, such as RNS therapy. The pivotal RNS trials established that patients whose seizures did not respond to VNS were as likely as those without prior VNS therapy to respond to RNS [7,8]. However, to the best of our knowledge, larger cohorts of patients with combined active RNS and VNS have not been investigated. Here we present a multicenter retrospective study assessing the safety and effectiveness of the concurrent use of VNS and RNS.

## 2. Methods

We performed a retrospective chart review on all patients treated with concurrent active VNS and RNS for a minimum of six months across ten comprehensive epilepsy centers. The adult patients had drug-resistant focal epilepsy and were treated according to the indication for use for each system. Each of the participating centers received approval from their institutional review boards to collect the following data from their patients’ charts: demographics, epilepsy history, presurgical workup, neurostimulator settings, and seizure frequency. We collected patient-reported frequency of disabling seizures, including focal aware with motor component, focal with impaired awareness, and focal to bilateral tonic-clonic seizures. The disabling seizure frequency was determined by the documented patient and caregiver’s self-report at pre-VNS placement (if available), pre-RNS placement, one-year post RNS placement, and at the most recent follow-up. The median percent reduction in frequency of disabling clinical seizures and the responder rate (≥50% reduction in frequency of disabling seizures) at one year and at last follow-up were calculated relative to the patient’s pre-RNS baseline.

Information regarding qualitative VNS or RNS effects on seizure severity or duration was not consistently documented and was therefore not gathered. Serious adverse events defined as requiring medical attention or hospitalization found to be related to the VNS and RNS were collected, though VNS implant data were not obtained in this study as these procedures were frequently done many years earlier and data very difficult to acquire. Patient-reported stimulation side effects were reviewed and documented based on clinic notes and physician recollection. Of note, acquisition and documentation of patient-reported side effects implied questioning limited to anticipated effects, e.g., for VNS, questions about dysphonia and cough would be asked, while RNS would prompt examiners to inquire about headache.

Vagus nerve stimulator model number, duty cycle, and amplitude settings were collected from the chart at the time of RNS placement, at one-year post RNS placement, and at last follow-up. The RNS stimulation charge density was collected from the online Patient Data Management System at one-year post RNS placement and at last follow-up.

Chi-squared proportion test was used to compare differences between responders (≥50% reduction in seizure frequency) and non-responders.

## 3. Results

### 3.1. Patient demographics and RNS lead locations

There were 64 patients included with *both active* VNS and RNS in this analysis across 10 centers. In all but one case VNS was placed before RNS. The demographics of this group are presented in Table 1. Mesial temporal structures were the most common seizure-onset zone on both scalp EEG in 51% (33/65), and during intracranial monitoring (ICM) in 48% (22/46) of cases. Neocortical foci were found most often in the temporal lobes, followed by frontal, parietal, and occipital lobes.

Forty-one percent (26/64) of patients had RNS leads placed in a mesial temporal lobe, 42% (27/64) had neocortical leads, 13% (8/64) had one lead placed in a mesial temporal lobe and a second lead placed in a second focus, and 5% (3/64) had one lead placed in the thalamus and another lead placed in a separate seizure focus. Fifty-eight percent (37/64) had only depth leads, 33% (21/64) had only cortical strip leads, and 9% (6/64) had a combination of depth and cortical strip leads connected to the neurostimulator. Fifty percent (32/64) of patients had bilateral targets, and 45% (29/64) had unilateral targets, while 5% (3/64) had thalamic targets.

Thirty percent (19/64) of patients did not have ICM. In these cases, RNS lead placement was determined by imaging and scalp EEG results. Seventy-nine percent of these (15/19) received mesial temporal RNS leads (11 patients with bilateral mesial temporal implantations, 1 with unilateral mesial temporal, 3 with one mesial temporal, and one other target (2 neocortical, 1 thalamus)), 16% (3/19) had neocortical RNS leads, and 5% (1/19) had bilateral thalamic leads. Fifteen of these patients had a lesional MRI, all 4 MRI-negative patients received bilateral mesial temporal RNS depth leads.

### 3.2. VNS treatment only (prior to RNS treatment)

Data on clinical seizure reductions with VNS prior to RNS placement were available for only 47% (30/64) of patients. The median duration of VNS treatment prior to RNS implantation was 47 months (mean: 58, SD: 42 months, range: 5–167 months). The median duration of VNS treatment to most recent follow-up was 73 months (mean: 83, SD: 37 months, range: 34–186 months). Given the small sample size for patients with pre- and post-VNS seizure frequency data and selection bias of those with less than optimal response to VNS for this study, analysis of seizure outcome on VNS alone was considered not meaningful.

### 3.3. RNS treatment in combination with VNS treatment

**Fig. 1.**
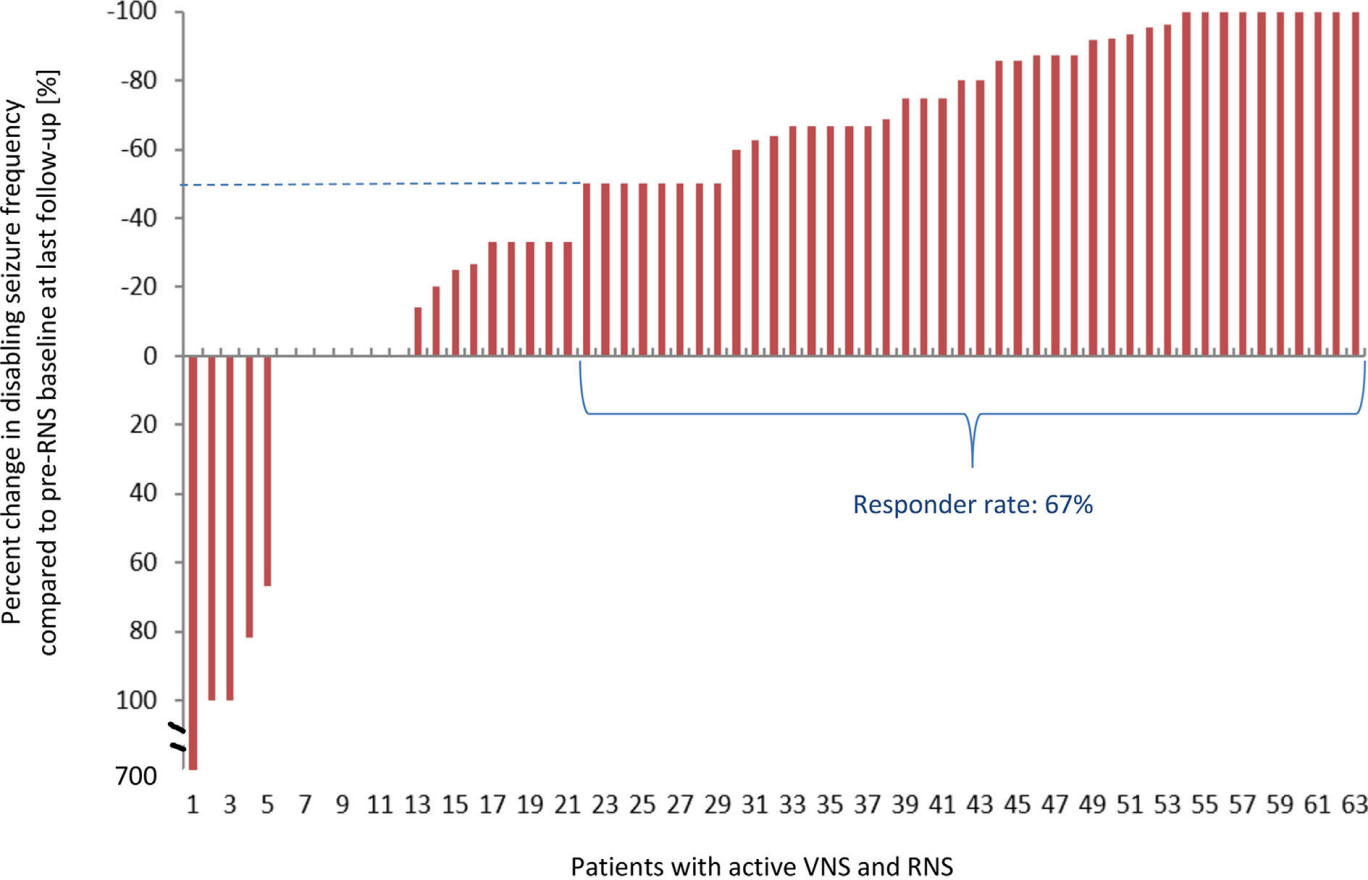
Change in clinically disabling seizures at last follow-up in all patients that had RNS placed after VNS with both systems active (*N* = 63).

Sixty-three patients had the RNS placed *after* the VNS. In these patients, median follow-up after RNS placement was 28 months (mean: 28 months, range: 6–56 months). Seizure reports were available for all 63 patients before RNS placement (baseline), at one year and at last follow-up after RNS placement. The median reduction in frequency of disabling seizures with concurrent VNS and RNS treatment at one year post RNS placement was 43% (mean: 26%, SD: 104%, range: decrease by 100% to increase by 700%) with a responder rate of 49%. The median reduction in seizure frequency at last follow-up was 64% (mean: 39%, SD: 106%, range: decrease by 100% to increase by 700%) and the responder rate was 67%. Nine patients (14%) reported being seizurefree at one year post RNS and 10 patients (16%) reported being seizurefree at last follow-up (Fig. 1).

One patient had the VNS placed 37 months after the RNS. With the RNS alone, the patient had experienced a 58% seizure frequency reduction at 1 year after RNS placement. With both systems used in combination for a 17-month period, the patient reported a 68% seizure frequency reduction at last follow-up, 54 months after RNS placement.

Stimulation settings for the VNS and RNS systems at all time points are shown in Table 2. Stimulation parameter setting for VNS and RNS were all within typical manufacturerrecommended ranges.

In 65% (42/64) of patients, the number of ASMs did not change after RNS implantation. Nineteen percent (12/64) of the patients had an increase in the number of ASMs, and 16% (10/64) had a decrease in the number of ASMs.

### 3.4. Safety

There were four types of adverse events reported after RNS implantation surgeries: (1) four soft tissue infections, (2) one skin erosion due to a seizure-related fall, (3) one asymptomatic subdural hematoma, and (4) one postoperative pseudomeningocele. All of these resolved without long-term neurological sequelae, and there was no need for device explantation. One patient (2%) reported stimulation-related side effects from RNS stimulation that involved disruption of language comprehension. This patient had two cortical strip leads placed over Wernicke’s area and the ability to perceive RNS stimulation was verified during in-office testing. The current amplitude and stimulation pathway were adjusted such that stimulation was no longer perceptible to the patient and the receptive language problems resolved.

Data on complications related to VNS implantation were not available. Thirty percent (19/64) of patients reported stimulation-related side effects related to VNS stimulation before and after RNS placement. The documented VNS stimulation side effects reported by patients at one year post RNS placement were: voice alteration (9%), cough/hoarseness (5%), throat tightness (3%), and sleep disruption (2%). At last follow-up VNS stimulation side effects reported by patients were: voice alteration (14%), cough/hoarseness (5%), and pain (2%).

In this patient cohort, there were no device–device interactions reported between the VNS and RNS systems. Specifically, artifact from VNS stimulation was not apparent in ECoGs captured by RNS, and there was no documentation of RNS stimulation-triggered VNS stimulation or effect on heart rate. There was no report of unintended effect of magnet use for one device on the other device.

One patient experienced severe depression and suicidal ideation necessitating hospitalization that the clinician identified as having an unclear relationship to the RNS or VNS systems. Responsive neurostimulator leads were placed in the left anterior hippocampus and the left middle hippocampus. The VNS in this patient had been turned off five months prior to RNS placement; it was turned back on prior to RNS, but the exact timing is unclear. The mood changes occurred soon after the RNS stimulation was enabled. Both VNS and RNS were stimulating when the depression and suicidal ideation resolved. One patient developed significant anxiety and insomnia one year after the RNS was placed (VNS was placed several years prior). Responsive neurostimulator leads were placed in both hippocampi. The symptoms worsened when the VNS and RNS were both switched off; therefore, these adverse events were not considered related to either system. The anxiety and insomnia are ongoing.

## 4. Discussion

To date, there are limited data on the safety and effectiveness of concurrent VNS and RNS therapy because participants in the clinical RNS trials had to have VNS explanted to be eligible [1]. The unique contribution of this study is to describe real-world clinical outcomes in patients with combined use of active VNS and RNS systems. This study was not powered to draw conclusions on the effectiveness of each system alone or in combination.

Concurrent use of the VNS and RNS appears to be safe in our cohort of 64 patients with drug-resistant focal epilepsy. There were no negative interactions observed between the two systems, and VNS stimulation artifact was not observed in RNS ECoGs, consistent with a previous report [3]. The retrospectively reported adverse event types and rates are similar to those in studies of the VNS or RNS devices in isolation [4,5]. In terms of stimulation-related side effects, 30% of patients experienced side effects related to VNS stimulation, most expected, minor, and often lessening with time, and one (2%) patient experienced RNS-stimulation-related side effects which were mitigated with changes to the device setting as previously described [9]. It is worth noting, however, that even though RNS stimulation is typically well-tolerated, there were some peri-implant adverse events, including intracranial hemorrhage, which would not be expected to be associated with VNS implantation. Additionally, there are no data in this cohort of patients pertaining to safety related to MRI with concurrent devices.

In 63 patients, the addition of RNS treatment to the already active VNS resulted in a 43% and 64% median seizure frequency reduction at 1 year and at last follow-up, respectively. These results are similar to those of RNS clinical trials which demonstrated a 44% median seizure frequency reduction at 1 year and 53% median reduction at 2 years [10]. However, the outcome results we observed are lower than those reported in the Real-World RNS study [2], of 67% at 1 year and 75% at 2 years. We postulate that the lower effectiveness could imply that our patient cohort, all with seizures relatively non-responsive to VNS, represent a group that is more treatment-resistant than the real-world cohort.

This patient cohort achieved a remarkable seizure frequency reduction, suggesting that patients with seizures that have a suboptimal response to VNS retain the potential to respond to therapy when active VNS is combined with RNS, although delayed effects of prolonged VNS therapy cannot be ruled out. The converse may also be true, but our population (*N* = 1) with VNS following RNS is too small to draw conclusions. While the pivotal RNS trial [7,8] already showed that patients with and without *prior* VNS treatment were equally likely to benefit from RNS, our findings support that the *combination* of these neuromodulation therapies is feasible, safe, and beneficial.

Although the precise therapeutic mechanisms of action of the VNS and RNS devices remain unclear, it is likely that VNS and RNS act upon different networks or levels within a seizure network, especially as it relates to long-term network effects [11,12]. Stimulation of the vagus nerve with the VNS is postulated to influence brainstem to thalamic connections, and RNS is thought to affect cortical, limbic, and thalamic connections. Individual patients could respond to one approach or the other, depending on their seizure network. In some patients, a combined approach could be most advantageous. DBS, which predominantly affects limbic circuitry, could also have an added or synergistic effect [6].

### 4.1. Limitations

The main limitation of our study is the retrospective nature of our sample and the relatively small population size. Although each patient is treated as their own control, a major limitation of the within-patient control is the preponderant unidirectionality of treatment sequence from VNS therapy to added RNS. There were no control groups for our population of patients with active VNS and RNS treatment.

Direct comparisons cannot be made between patient cohorts with VNS treatment only and those with the addition of the RNS as this study was not designed to answer this question.

Another limitation is that although the pre- and postimplantation data were available for all 64 patients who had the RNS device placed, they were only available for 47% of patients before and after VNS placement, making it difficult to determine VNS effect on seizure frequency. Recall bias regarding estimating seizure frequency by patient or caregivers could also present a limitation, although this would likely be the case across all groups of patients. In addition, there was no reliable information available with respect to the VNS or RNS effect on seizure severity.

The VNS device has undergone modifications with each model. Although we were able to determine that at last follow-up 30% of patients had a VNS model with ictal tachycardia detection and the median current setting was 1.5 mA on low duty cycle, given the small sample size, it was not possible to control for adjustments made to the VNS settings. Additionally, information pertaining to changes in VNS settings was not obtained and it is possible that VNS settings were altered.

One of the limitations as it pertains to documentation of adverse events is related to how patient-reported side effects are elicited by physicians, with physicians tailoring questions depending on which device was implanted. Retrospective chart review depends on the accuracy of documentations and may underestimate adverse effects.

Although 63% of patients had no changes to the number of ASMs during this study period, another limitation is that ASMs were not held constant during this time period. As in other uncontrolled, retrospective studies, the effect of medication adjustments cannot be accurately assessed and therefore was not taken into account in reporting change in seizure frequency.

### 4.2. Conclusion

Despite the study’s limitations, we find it appears safe to use neuromodulation devices, specifically VNS and RNS concurrently. Lack of seizure response to one device does not necessarily predict failure to treat seizures more effectively by adding another. Patients are already being treated with more than one neurostimulation device and our data support this practice. Clinicians should consider combining different neuromodulation modalities if the first device provided no or only minimal improvement of seizure frequency. The effectiveness of different neurostimulators when used in combination probably relates to unique mechanism of action for each device, but further research is necessary to elucidate such mechanisms and tailor use of neuromodulation options to each patient. We have answered some preliminary questions regarding safety and effectiveness of the concurrent use of VNS and RNS; however, randomized controlled studies are needed in the future to address the potential treatment synergies between these two forms of neuromodulation and to compare directly the outcomes of patients treated with each system alone and in combination.

## Figures and Tables

**Table 1:** Patient demographics.

	All Patients *N* = 64

**Gender Female [*N*] (%)**	33 (52)
**Mean Age [years ± SD] (range)**	36.0 ±11.8 (18–68)
**Mean Age at Dx [years ± SD] (range)**	12.4 ± 9.9 (0–44)
**Median Baseline Disabling Seizures per month [*N* szs] (IQR)**	8 (4–16)
**Mean ± SD (range)**	20.8 ± 44.6 (0.33–300)
**[*N*]**	63
**Etiology** [*N*] (%)	
***Structural***	27 (42)
***Genetic***	1 (2)
***Metabolic***	0 (0)
***Infectious***	5 (8)
***Immune***	2 (3)
***Unknown***	21 (33)
***Other***	8 (13)
**MRI [*N*] (%)**	
***Abnormal***	50 (78)
***Normal***	14 (22)
**Prior Intracranial Monitoring [*N*] (%)**	45 (70)
**Intracranial localization [*N*] (%)**	
***Bilateral Single Lobe***	13 (29)
***Bilateral Multilobar***	3 (7)
***Unilateral Single Lobe***	12 (27)
***Unilateral Multilobar***	15 (33)
***Unilateral Other***	2 (4)
**Prior Epilepsy Surgery [*N*] (%)**	32 (50)

Dx: diagnosis; IQR: interquartile range; [*N*]: number of patients; [*N* szs]: number of seizures; SD: standard deviation; SD: standard deviation.

**Table 2:** Device stimulation settings at each time point.

System	Baseline (at RNS implant)	1 year	Last Follow-up

**VNS** Model	*N* = 53	*N* = 51	*N* = 51
Aspire (ictal tachycardia detection)	23%	22%	30%
**VNS** Current [mA]	*N* = 62	*N* = 57	*N* = 56
Median	1.5	1.6	1.5
Mean	1.8	1.7	1.7
Range	0.25–3.5	0.25–3.25	0.5–3.25
**VNS** Duty Cycle	*N* = 60	*N* = 56	*N* =53
Patients with Low duty cycle	82%	87%	90%
**RNS** charge density [μC/cm^2^]	Stimulation not enabled	*N* = 44	*N* = 45
Mean		2.19	2.73
SD		±0.78	±1.18
Range		0.8–3.8	0.5–6.1

Note: Due to the retrospective nature of the study, not every data point could be collected for each patient. Low duty cycle was defined as ≤35% (Duty Cycle = (ON Time + 4 s)/(ON time + OFF Time), for which ON and OFF Times are measured in seconds; duty cycle increases when ON times increase and/or OFF times decrease).
